# Ampicillin-treated *Lactococcus lactis* MG1363 populations contain persisters as well as viable but non-culturable cells

**DOI:** 10.1038/s41598-019-46344-z

**Published:** 2019-07-08

**Authors:** Rinke J. van Tatenhove-Pel, Emile Zwering, Ana Solopova, Oscar P. Kuipers, Herwig Bachmann

**Affiliations:** 1 Systems Bioinformatics, Amsterdam Institute for Molecules, Medicines and Systems, VU University Amsterdam, de Boelelaan 1108, 1081 HV, Amsterdam, The Netherlands; 2 NIZO Food Research, Kernhemseweg 2, 6718 ZB Ede, The Netherlands; 3 Department of Molecular Genetics, Groningen Biomolecular Sciences and Biotechnology Institute, University of Groningen, Nijenborgh 7, 9747 AG, Groningen, The Netherlands

**Keywords:** Physiology, Bacteria

## Abstract

*Lactococcus lactis* is used as cell-factory and strain selections are regularly performed to improve production processes. When selection regimes only allow desired phenotypes to survive, for instance by using antibiotics to select for cells that do not grow in a specific condition, the presence of more resistant subpopulations with a wildtype genotype severely slows down the procedure. While the food grade organism *L. lactis* is not often exposed to antibiotics we characterized its response to ampicillin in more detail, to better understand emerging population heterogeneity and how this might affect strain selection procedures. Using growth-dependent viability assays we identified persister subpopulations in stationary and exponential phase. Growth-independent viability assays revealed a 100 times larger subpopulation that did not grow on plates or in liquid medium, but had an intact membrane and could maintain a pH gradient. Over one third of these cells restored their intracellular pH when we induced a temporary collapse, indicating that this subpopulation was metabolically active and in a viable but non-culturable state. Exposure of *L. lactis* MG1363 to ampicillin therefore results in a heterogeneous population response with different dormancy states. These dormant cells should be considered in survival-based strain selection procedures.

## Introduction

*Lactococcus lactis* is an important organism in the dairy industry and it is increasingly explored as a cell factory for food ingredients and as a delivery vehicle for bioactive molecules^1,2^. To improve the yield or titer of the compounds of interest or to decrease the amount of by-products, strain selections are regularly performed^3,4^. In these selections heterogeneity in the single cell response is relevant, especially in dominant selection systems in which mutants are selected based on the principle that only cells with a desired phenotype can survive^5^. If resistant subpopulations with a wild-type genotype also survive the imposed condition, they will severely slow down the selection process.

An example is that *L. lactis* produces high concentrations of lactate during food fermentations resulting in a low final pH, which is not always desired. We initially sought to select mutants that do not acidify the growth medium below a certain pH and intended to achieve this by simply exposing a partly acidified culture to ampicillin, thereby killing all cells that can grow in these conditions. However, we found high fractions of surviving cells which completely acidified the medium after re-growth, and which were not ampicillin resistant. *L. lactis* is a food grade organism and therefore not often exposed to ampicillin. Nevertheless, we characterized its response to ampicillin in more detail, to better understand the heterogeneity in its population response and how this might affect strain selection procedures.

When a bacterial population is exposed to a bactericidal antibiotic like ampicillin, most cells will die^6^. To survive the antibiotic treatment cells can acquire resistance through e.g. export of the antibiotic using multidrug-resistance pumps or through the expression of antibiotic degrading enzymes^7^. However, dormant, non-growing cells can also survive treatments with antibiotics that target growth-related processes^8,9^. These dormant cells can revive spontaneously or when conditions change, and therefore they play a role in chronic infections^10–14^. Two well-known dormancy states are the persister state and the viable but non-culturable (VBNC) state^15^.

Persisters were first described in 1944 by Joseph Bigger^16^. He showed that penicillin could not completely kill growing staphylococcal cultures and that surviving cells were as sensitive to penicillin as the parent culture. The addition of an antibiotic to a population with persisters results in bi-phasic killing curves, because growing cells die quickly while persisters die only when they spontaneously switch back to the growing state^17–19^. Persister subpopulations have been identified in several species, including *Escherichia coli*, *Pseudomonas aeruginosa*, *Staphylococcus aureus*, *Candida albicans*, *Enterococcus faecalis* and *Mycobacterium tuberculosis*^12,20–22^.

VBNC cells are metabolically active, but non-growing cells. Their presence is reported in over 100 species^15,23^. The VBNC state is induced by environmental stresses, but viable non-culturable cells are also reported to be stochastically present in cultures^24,25^. They can coexist with persisters and a dormancy continuum was suggested, in which cells first enter the persister state and thereafter the VBNC state^24^. A limited amount of studies analyzed the presence of VBNC cells in *L. lactis* populations. VBNC cells were found under carbohydrate starvation, pH stress and during retentostat cultivation^26–29^. To our knowledge their presence is not reported under any other (stress) conditions.

Unlike persisters, VBNC cells do not grow on agar plates or in liquid medium unless they are resuscitated^24^. To detect VBNC cells, growth-independent viability assays are used, which measure for instance membrane integrity or enzyme activity^30,31^. Another growth-independent viability criterion is the ability to maintain a pH gradient, which requires an intact cell membrane and metabolic activity^32^. The intracellular pH can be measured using intracellular compounds with a pH dependent fluorescence signal, such as fluorescein-based stains or fluorescent proteins like GFP^32–35^.

Here we characterized the response of *L. lactis* MG1363 to ampicillin. CFU counting revealed bi-phasic killing curves, indicating that *L. lactis* MG1363 forms persisters. LIVE/DEAD staining and GFP-based intracellular pH measurements showed that in addition to the persister fraction 100 times more cells were in a VBNC state after exposure to ampicillin. Given the observed heterogeneity in the single cells response, we discuss how the efficiency of dominant selection systems can be improved.

## Materials and Methods

### Construction of *L. lactis* MG1363_GFP

The gene coding for the green fluorescent protein (Dasher-GFP) was obtained from ATUM (previously DNA 2.0; Newark, CA, USA) in a pJ221-based *E. coli* vector. The *gfp* gene was inserted in *Lactococcus lactis* MG1363 integration vector pSEUDO::P*usp45*-sfgfp(Bs)^36^ as an XhoI/BcuI restriction fragment replacing the resident *sfgfp(BS)* gene. The resulting vector pSEUDO::P*usp45*-*gfp* was maintained in *E. coli* DH5α. *L. lactis* MG1363_GFP was obtained by a single-crossover integration of pSEUDO::*Pusp45-gfp* into the *pseudo 10* locus on the chromosome of *L. lactis* MG1363. Integration was performed as described previously^36^.

### Strains and media

**Figure 1:**
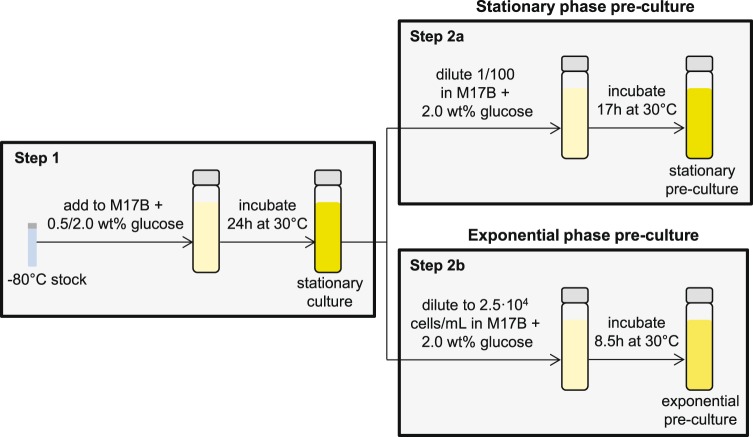
Overview of the pre-culture procedure. In step 1 2.0 wt% glucose was used for the stationary phase pre-culture and 0.5 wt% glucose for the exponential phase pre-culture.

*L. lactis* MG1363^37^ and *L. lactis* MG1363_GFP were cultured in M17 broth supplemented with 2.0 wt% glucose, unless otherwise indicated. M17 supplemented with 1.0 wt% glucose and 1.1 wt% agarose was used for plate counting. Cultures and plates were incubated at 30 °C. As suggested by Harms *et al*. we standardized the pre-culture procedure (Fig. 1) and used exponential phase pre-cultures in balanced growth (13 generations in excess glucose)^38^. 10 or 100 µg/mL ampicillin (Applichem, Darmstadt, Germany) was directly added to exponential pre-cultures; stationary pre-cultures were diluted 1/100 in fresh medium with ampicillin. Heat-killed cells were prepared by incubating stationary pre-cultures in phosphate buffered saline (PBS) of pH 7.5 at 80 °C for 20 minutes. *E. coli* DH5α was used for cloning and grown in LB medium at 37 °C with shaking or on LB medium solidified with 1.5 wt% agar supplemented with 100 µg/mL erythromycin.

### Growth assays

To measure if cells could grow samples were centrifuged for 2 minutes at a relative centrifugal force (rcf) of 16,000 g. Pellets were resuspended and diluted in PBS (pH 7.5) and plated in duplicate. Plate counts were determined after at least 48 hours of incubation. Alternative growth assays were done in liquid medium using the most probable number (MPN) procedure. 16 replicates of a 10-fold dilution series were scored for growth after 7 days of incubation. MPN counts were determined as described earlier^39^.

### Flow cytometry measurements

An Accuri C6 flow cytometer (BD Biosciences, San Jose, CA, US) was used for flow cytometry measurements.

To determine the number of intact cells we used the LIVE/DEAD BacLight bacterial viability kit L7007 (Molecular Probes, Eugene, OR, US) according to the manufacturer’s instructions for flow cytometry measurements. To determine the thresholds to separate live- and dead-stained cells a calibration curve was prepared with mixtures of living and heat- or ethanol-killed cells.

For intracellular pH measurements we centrifuged samples for 2 minutes at an rcf of 16,000 g and we resuspended the pellets in PBS with 0.5 wt% glucose at a desired pH (4.0–8.0). Glucose was added to keep the cells in an energized state, allowing metabolically active cells to maintain a pH gradient^40^. Below pH 5.5 PBS has a low buffering capacity, we therefore monitored the pH of the samples to ensure that it was constant. Cells were uncoupled by adding 1 µM valinomycin (a K^+^ transporter) and 2 µM nigericin (a H^+^ K^+^ antiporter) to the sample, followed by an incubation for 5 minutes at room temperature. Valinomycin and nigericin together cycle K^+^ and transport H^+^, ensuring that the intracellular pH equals the buffer pH^40,41^. To determine which cells could maintain a pH gradient we set a fluorescence threshold based on the fluorescence of uncoupled cells measured at pH 7.0.

### Persister-model fit

We fitted the persister model of Balaban *et al*. to our experimental data^17^. The persister percentage at t = 0 h was estimated by extrapolating the second phase of the killing curve, in which persisters switch to the growing state, to t = 0 h.

## Results

### Membrane pH gradients can be determined using GFP

**Figure 2:**
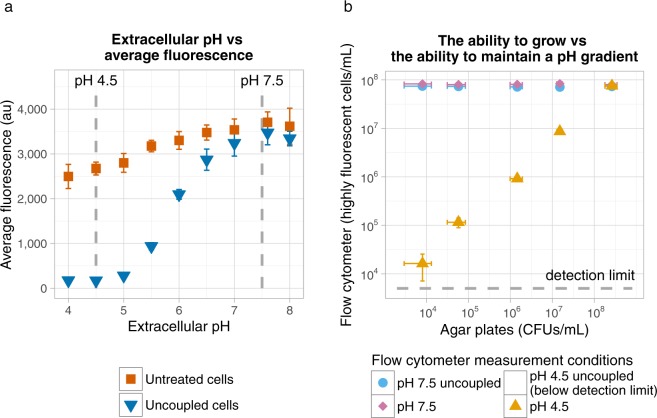
(**a**) GFP signal of *L. lactis* MG1363 at different extracellular pH values. Stationary *L. lactis* MG1363_GFP cells were measured before and after uncoupling their pH gradient. Some error bars (SEM, n = 3) are too small to be visible. **(b**) Correlation between the ability to grow and the ability to maintain a pH gradient. For mixtures of living (stationary) and heat-killed cells we measured the ability to grow on agar plates and the ability to maintain a pH gradient by looking at their GFP fluorescence at pH 4.5. As controls we measured the fluorescence at pH 7.5 and the fluorescence after uncoupling the pH gradient. Some error bars (SD, n = 3) are too small to be visible.

Metabolically active *L. lactis* cells are reported to maintain a pH gradient over their membrane: at low extracellular pH they can keep their intracellular pH above the extracellular pH^40,42^. The intracellular pH can be measured with GFP, as its fluorescence intensity decreases with a decreasing pH^33,34^. To test if cells that maintain a pH gradient over their membrane show a higher GFP signal than cells without a pH gradient, we compared the fluorescence of stationary *L. lactis* MG1363_GFP cells with cells in which the pH gradient was dissipated by valinomycin and nigericin (uncoupled cells). At pH 7.5 we observed that both uncoupled and untreated stationary cells were highly fluorescent, while at pH 4.5 uncoupled cells gave a 15 times lower fluorescence signal than untreated cells (Fig. 2a).

To compare the ability to grow and the ability to maintain a pH gradient, we measured mixtures of living and heat-killed cells. At pH 7.5 living and heat-killed cells showed a high fluorescence signal, indicating that the heat-treatment did not denature the GFP and cells did not lyse (Fig. 2b). At pH 4.5 we found no significant difference between the number of highly fluorescent cells per mL and the CFUs per mL at different ratios of living and heat-killed cells (paired t-test, p = 0.56). We subsequently evaluated whether highly fluorescent cells indeed maintained a pH gradient by adding valinomycin and nigericin to the cells. At pH 7.5 uncoupling did not affect the number of highly fluorescent cells per mL, but at pH 4.5 it dropped below the detection limit. These results confirm that highly fluorescent cells at pH 4.5 maintained a pH gradient. Together these results show that for stationary and heat-killed *L. lactis* MG1363 the presence of a pH gradient correlated well with the cell’s ability to grow.

### Ampicillin-treated *L. lactis* MG1363 cultures show bi-phasic killing curves

**Figure 3:**
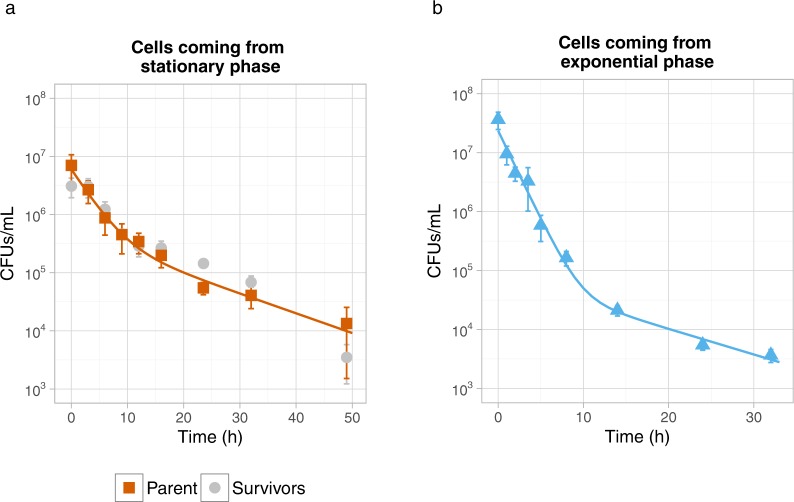
Killing curves of ampicillin treated *L. lactis* MG1363. Stationary **(a)** and exponential **(b)** *L. lactis* MG1363 cells were diluted in medium with ampicillin and their ability to grow was measured on agar plates. Surviving cells from culture (**a**) (Parent) were propagated in medium without ampicillin and a second killing curve was recorded (Survivors). Some error bars (SD, n = 3) are too small to be visible. The solid line indicates the fit of a persister-model^17^.

Exposing *L. lactis* MG1363 to ampicillin showed that a considerable fraction of the cells survived. We subsequently determined the CFU counts in time and found bi-phasic exponential killing curves (Fig. 3). After 48 hours of ampicillin exposure we re-grew the survivors and exposed them again to ampicillin, to determine if these cells were mutants that acquired ampicillin resistance. Re-grown survivors also showed bi-phasic killing (Fig. 3a), indicating that the bi-phasic behavior was not caused by genetic changes, but rather by phenotypic heterogeneity. We fitted the persister model of Balaban *et al*. to our data^17^. Based on the model the estimated persister fractions were 7.6 ± 3.6% in a stationary phase culture and 0.32 ± 0.17% in an exponential phase culture.

### Presence of toxin/antitoxin systems in *L. lactis* MG1363

Previous studies proposed toxin/antitoxin (TA) systems as a mechanism for persister formation^43^. The TA database TADB2.0 predicts five type II TA pairs in *L. Lactis* MG1363^44^, from which two are 100% identical at the protein level. The predicted toxins contain Xre domains, the predicted antitoxins contain RelE, COG2856 and Bro domains. A phylogenetic analysis with experimentally validated TA proteins retrieved from the TADB2.0 database was performed^44^. This analysis showed that the *L. lactis* MG1363 proteins fall within clades of experimentally validated TA systems. However, sequence identities for many of the proteins are low (typically <25%) and it therefore remains unclear if these predicted TA systems are active in *L. lactis* MG1363.

### Ampicillin-treated *L. lactis* MG1363 cultures contain viable but non-culturable cells

**Figure 4:**
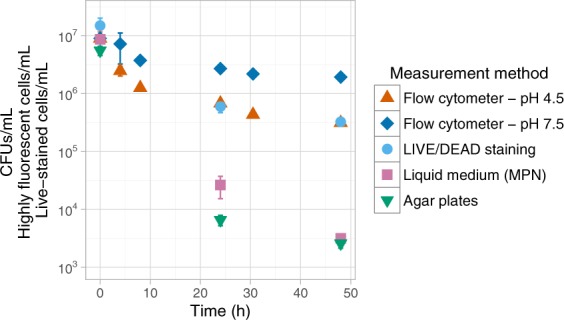
Growth and maintenance of a pH gradient in ampicillin treated *L. lactis* MG1363. We diluted stationary *L. lactis* MG1363_GFP cells in medium with ampicillin and measured their ability to grow in liquid medium and on agar plates (shown as CFUs/mL) and their ability to maintain a pH gradient in time (shown as highly fluorescent cells/mL). LIVE/DEAD staining was used to determine the amount of *L. lactis* MG1363 cells that have an intact membrane after ampicillin exposure (shown as live-stained cells/mL). Some error bars (SD, n = 3) are too small to be visible.

To determine the viability of ampicillin-treated *L. lactis* MG1363 with a growth-independent assay we measured the GFP fluorescence of cells and analyzed if they could maintain a pH gradient. We only analyzed the response to ampicillin for cells coming from stationary phase, because the persister fraction in exponential cultures is low and we approached the detection limit of our GFP-based assay under these conditions. After 48 hours of exposure to ampicillin the concentration of highly fluorescent cells in the cell suspension at pH 7.5 was reduced to 22 ± 4% compared to the start of the experiment, suggesting that 78% of the cells lysed (Fig. 4). Within this 22 ± 4% two subpopulations were present. 18 ± 3% of the cells were highly fluorescent at pH 7.5, but not at pH 4.5. These cells could not maintain a pH gradient, similar to what was observed for the heat-killed cells in Fig. 2b. The remaining 3.5 ± 0.8% of the cells showed high fluorescence also at pH 4.5. These cells could maintain a pH gradient, indicating that they had an intact membrane and suggesting that they were metabolically active. However, only 0.029 ± 0.004% of the cells could grow on agar plates, suggesting that cells entered the VBNC state. We tested four alternative hypotheses that could explain why we found 100-fold more cells with a high fluorescence signal than cells that could grow: (1) the GFP-based assay over-estimates the number of cells with an intact membrane, (2) the used ampicillin concentration is too low to completely kill all dividing cells, (3) highly fluorescent cells can grow, but not on agar plates, 4) highly fluorescent cells are dead, but they have a high intracellular pH because their membrane is still intact.

To independently confirm the results of the GFP-based assay we also performed LIVE/DEAD staining on cells that were exposed to ampicillin. As *L. lactis* MG1363_GFP is already highly fluorescent we used its parental strain *L. lactis* MG1363 for this assay. After 24 and 48 hours of ampicillin exposure the number of live-stained cells was similar to the number of cells that could maintain a pH gradient (Fig. 4), confirming that the fraction of cells with an intact membrane is much higher than the number of cells that can grow.

The second option considered was that the used ampicillin concentration was too low to completely kill all dividing cells. We hypothesized that increasing the ampicillin concentration would lower the number of cells that maintained a pH gradient, bringing it to the level of the number of cells that could grow. To test this hypothesis we diluted stationary *L. lactis* MG1363_GFP cells in medium supplemented with 100 µg/mL instead of 10 µg/mL ampicillin, and measured their ability to grow and to maintain a pH gradient. After 48 hours of exposure to 100 µg/mL ampicillin the difference between the number of cells that maintained a pH gradient and the number of cells that could grow was larger than it was for 10 µg/mL ampicillin (10 µg/mL ampicillin: 3.5 ± 0.8% maintained a pH gradient and 0.029 ± 0.004% could grow; 100 µg/mL ampicillin: 15 ± 1% maintained a pH gradient and 0.035 ± 0.016% could grow). These results showed that increasing the ampicillin concentration did not reduce the difference between the number of cells that maintained a pH gradient and the number of cells that could grow, it even increased the difference. This indicates that the observed 100-fold difference in the ability to grow and the ability to maintain a pH gradient is not caused by using a too low ampicillin concentration.

The third option considered was that cells that maintained a pH gradient had difficulties growing on agar plates. However, after 48 hours of exposure to ampicillin we found no significant difference in growth on agar plates and in liquid medium (Fig. 4), confirming that cells that maintained a pH gradient were unable to grow on both agar plates and in liquid medium.

**Figure 5:**
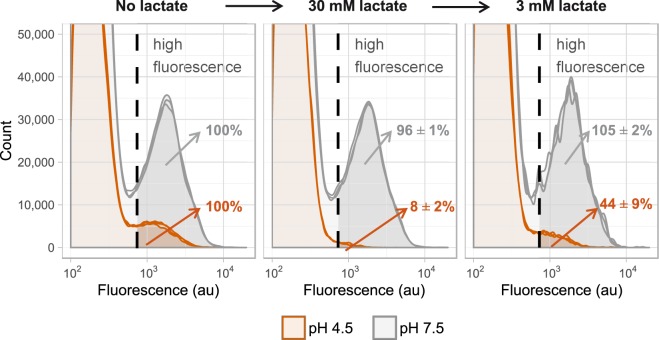
Metabolic activity of ampicillin treated *L. lactis* MG1363. Stationary *L. lactis* MG1363_GFP cells were diluted in medium with ampicillin (n = 3). After 48 hours (left panel) cells were temporarily exposed to high lactate levels (middle panel). At pH 4.5 this reduced the GFP fluorescence due to weak acid uncoupling. When the lactate concentration was lowered the amount of highly fluorescent cells increased from 8 ± 2% to 44 ± 9% (right panel). This shows that 36 ± 7% of the cells could restore the high fluorescence signal, indicating that these cells were metabolically active.

The fourth option considered was that the membrane of ampicillin-treated cells was intact, while the cells were actually dead. To test if the cell wall of the cells was damaged we placed ampicillin-treated cells in demineralized water for 2.5 hours, but the number of cells with an intact membrane remained constant (data not shown). If cells were only weakly permeable to protons this would allow metabolically inactive cells to have a high fluorescence signal at pH 4.5, because the cytosol is not yet acidified before the cells are measured. To test this hypothesis we analyzed in three steps if cells that were exposed to ampicillin for 48 hours could restore their pH gradient after temporary exposure to a high lactate concentration (Fig. 5). In the first step the GFP signal was measured in absence of lactate and the observed number of highly fluorescent cells was set to 100%. At pH 4.5 these are the cells that can maintain a pH gradient. In the second step 30 mM lactate was added to the sample. At pH 4.5 this led to weak acid uncoupling: the intracellular pH became similar to the extracellular pH and 92 ± 2% of the cells lost their high fluorescence signal. When the lactate concentration was diluted from 30 mM to 3 mM in the third step, the number of highly fluorescent cells increased from 8 ± 2% to 44 ± 9%, indicating that 36 ± 7% of the cells that maintained a pH gradient at pH 4.5 could restore their intracellular pH. When cells were not treated with ampicillin all cells restored their pH gradient after the lactate exposure (data not shown). Overall these results indicate that around 1.2% (36% of 3.5%) of the ampicillin-treated cells were metabolically active while only 0.029 ± 0.004% of the cells could grow, confirming that ampicillin-treated *L. lactis* MG1363 cultures contain VBNC cells.

**Figure 6:**
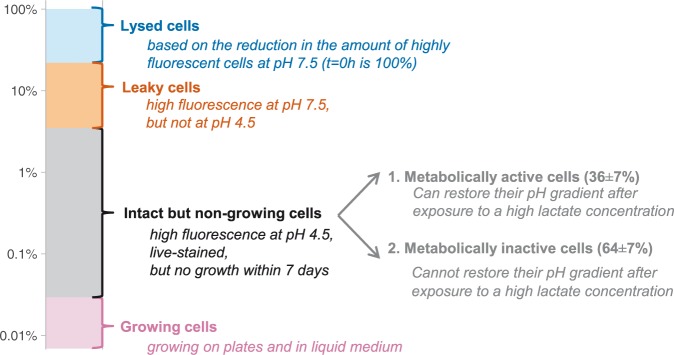
Schematic overview of the response of *L. lactis* MG1363 to ampicillin. Stationary *L. lactis* MG1363 or MG1363_GFP cells were diluted in medium with ampicillin. After 48 hours of ampicillin exposure we analyzed their viability by looking at their membrane integrity, their ability to grow in liquid medium and on agar plates, and their ability to maintain and build-up a pH gradient. This figure summarizes the combined results of these assays.

In total we used four different viability assays to analyze the response of *L. lactis* MG1363 to ampicillin. Figure 6 summarizes results obtained after 48 hours of ampicillin exposure.

## Discussion

Dormant bacterial cells can survive treatments with antibiotics that target growth-related processes and they can therefore cause chronic infections and food spoilage. In a different context they can influence mutant selections. In this study we analyzed the response of *L. lactis* MG1363 to ampicillin using four different viability assays, which led to the identification of several subpopulations (Fig. 6).

To our knowledge this is the first study that shows that *L. lactis* MG1363 can form a persister subpopulation. Harms *et al*. showed that antibiotic treatments can induce prophages, resulting in cell-lysis due to phages instead of the antibiotic^38^. The strains used in this study are “phage-cured”: they contain 2 prophages and 4 prophage remnants, but induction has not been reported^37^. We therefore do not expect that phage induction plays a role in our experiments.

We observed that after an ampicillin treatment only 0.029% of the cells could grow in liquid medium or on agar plates, while 1.2% showed metabolic activity based on GFP fluorescence measurements (Fig. 6). These findings indicate the presence of VBNC cells in ampicillin-treated *L. lactis* MG1363 populations. Previous studies found VBNC *L. lactis* cells during carbohydrate starvation, pH stress and in retentostat cultivations^26–29^. Our results show that exposure of *L. lactis* to ampicillin can also lead to VBNC cells. From 24 to 48 hours of ampicillin exposure we observed a decrease in the number of cells that maintained a pH gradient (Fig. 4), indicating that the number of VBNC cells decreased. Because VBNC cells do not spontaneously switch back to the growing state this decrease cannot be caused by killing of the cells by ampicillin^15^, but by a so far unidentified mechanism.

When *L. lactis* MG1363 is exposed to ampicillin it shows a heterogeneous population response with several dormancy states. Coexistence of persisters and VBNC cells is also described in *E. coli* and *Vibrio vulnificus*^24,25^. Toxin/antitoxin (TA) systems were for a long time widely accepted to play a role in persister formation and Ayrapetyan *et al*. proposed a dormancy continuum: with increasing toxin to antitoxin ratios cells first become persisters and then viable but non-culturable^43^. However, recent studies found no direct correlation between TA systems and bacterial persistence, and the role of these modules is therefore unclear at this moment^38,45–47^. In *L. lactis* AbiQ is reported to be a type III TA system, but this gene is located on a native *L. lactis* plasmid and is therefore not present in the plasmid-free *L. lactis* MG1363 used here^48^.The TA database TADB2.0 predicted five type II TA pairs in *L. lactis* MG1363^44^, but it remains to be determined if they are functional.

Next to TA systems the ppGpp-mediated stringent response is reported to play a role in persister formation in Gram-negative *E. coli* and *P. aeruginosa*^18,38,49,50^. In *E. coli* high ppGpp levels are also reported to induce the VBNC state^51^. In a dormancy continuum ppGpp could therefore be a trigger for both the persister and the VBNC state in Gram-negatives. For Gram-positives this connection is less clear. In the Gram-positive *S. aureus* Conlon *et al*. found no effect of the stringent response on persister formation^46^, and to our knowledge there is no correlation reported between ppGpp and the VBNC state in Gram-positives. In the Gram-positive *L. lactis* high ppGpp concentrations are reported to increase acid resistance^52^, but the effect on antibiotic resistance, persister formation and the VBNC state is unknown.

Recent studies report that in Gram-positives and Gram-negatives persisters are formed when the intracellular ATP concentration of cells drops^46,47^. However, in VBNC cells ATP levels are reported to be similar or higher than in culturable cells^53–56^. This difference in ATP concentration in persister and VBNC cells contradicts the dormancy continuum and points towards different mechanisms behind the persister state and the VBNC state. In this study we observed that after 48 hours of ampicillin exposure 1.2% of the cells could quickly restore their intracellular pH when we induced a temporary pH decrease due to weak acid uncoupling (Fig. 5). Lactic acid bacteria have three main systems to remove protons from their cytosol and increase their intracellular pH: ATPase proton pumping, arginine deiminase activity that results in production of the base NH_3_, and glutamate decarboxylase activity that results in proton consumption in the cytoplasm^57,58^. In our experiment *L. lactis* MG1363 restored its intracellular pH in absence of arginine and glutamate and in presence of glucose, leaving ATPase proton pumping as the only mechanism to increase their intracellular pH. Our results therefore indicate that cells that restored their intracellular pH were not ATP depleted. The cells that had a high fluorescence before the lactate treatment, but could not restore their intracellular pH might be cells with a low ATP content or dead cells with an intact membrane. Because our assay cannot distinguish between these two states and because the persisters are 100 fold less abundant than the VBNC cells, we cannot draw conclusions about the ATP content of persisters. Our data does however suggest that VBNC *L. lactis* MG1363 cells are not ATP depleted, similar to what was shown for different organisms in previous studies^53–56^.

In this study we looked at the response of *L. lactis* MG1363 to ampicillin. β-lactam antibiotics like ampicillin disturb the balance between peptidoglycan synthesis and hydrolysis in growing cells, resulting in cell lysis^59,60^. In VBNC cells the peptidoglycan structure is changed and induction of peptidoglycan hydrolysis plays a role in the resuscitation of these cells^30,61^. Because ampicillin targets processes related to the VBNC state, there could be a direct relation between ampicillin and the presence of VBNC cells. We also exposed *L. lactis* MG1363 to heat-stress in this study, and we found no significant difference between the number of cells that maintained a pH gradient over their membrane and the number of CFUs (Fig. 2), indicating that *L. lactis* MG1363 does not form VBNC cells in response to heat-stress. It would be interesting to expose *L. lactis* MG1363 to other stress conditions in future studies and analyze the presence of persisters and VBNC cells, to see if our observations are specific for ampicillin or part of a more general stress response.

An antimicrobial that is produced by *L. lactis* itself is the peptide nisin, which kills Gram-positive bacteria by inhibiting cell-wall biosynthesis and by forming pores in the membrane^62,63^. Nisin has the GRAS status and it is commonly used as a food preservative because several pathogenic bacteria like *Listeria monocytogenes* are nisin sensitive^64^. Nisin is reported to kill *S. aureus* persisters^65^, but *L. monocytogenes* forms nisin-tolerant persisters^66^. To our knowledge there are no studies looking at the effect of nisin on the VBNC state in bacteria. *L. lactis* MG1363 could be a good model organism to study the effect of nisin on dormancy, because this strain is nisin sensitive and it can form both persisters and VBNC cells.

This study shows that *L. lactis* MG1363 forms persister and VBNC cells in stationary and exponential phase. The presence of these dormant subpopulations can reduce the efficiency of survival-based strain selections for microbial cell-factories. In *L. lactis* MG1363 the observed persister fraction is significantly lower at a high growth rate (exponential growth on glucose) compared to the stationary phase, similar to what is observed in other studies on persistence^17,67^. This result matches previous observations that at a low growth rate cells are more stress-resistant and prepared for changes in the environment^68,69^. We therefore expect that low efficiency of survival-based selection strategies through culture heterogeneity can be prevented by using fast growing cells.

VBNC cells themselves could also be interesting as cell-factories. These cells are non-growing, but they are reported to be metabolically active, to express genes and to exchange compounds with their environment^31^. These cells might therefore give high product yields, because growth and product formation are uncoupled and there is no carbon loss to biomass, similar to retentostats^70,71^.

VBNC cells can survive antimicrobial treatments because they are dormant, but they can start growing when conditions change^30,61,72^. Because VBNC cells do not spontaneously grow, viability measurements using agar plates might underestimate the potential viability of a culture, which is relevant in food safety and during antibiotic treatments. In those cases viability should not only be assessed by looking at growth, but also by looking at metabolic activity.

## Data Availability

The datasets generated during the current study are available from the corresponding author on reasonable request.
